# Energy Loss Reduction for Distribution Electric Power Systems with Renewable Power Sources, Reactive Power Compensators, and Electric Vehicle Charge Stations

**DOI:** 10.3390/s25071997

**Published:** 2025-03-22

**Authors:** Le Chi Kien, Tran Duc Loi, Minh Phuc Duong, Thang Trung Nguyen

**Affiliations:** 1 Faculty of Electrical and Electronics Engineering, Ho Chi Minh City University of Technology and Education, Ho Chi Minh City 700000, Vietnam; kienlc@hcmute.edu.vn (L.C.K.); loitd@hcmute.edu.vn (T.D.L.); 2 Power System Optimization Research Group, Faculty of Electrical and Electronics Engineering, Ton Duc Thang University, Ho Chi Minh City 700000, Vietnam; duongphucminh@tdtu.edu.vn

**Keywords:** electrical charging station, distribution electric power system, renewable energy sources, shunt capacitors, meta-heuristic algorithms, Chameleon Swarm Algorithm, Snow Geese Algorithm

## Abstract

This paper applies the Chameleon Swarm Algorithm (CSA) and Snow Geese Algorithm (SGA) for optimizing the placement of electric vehicle charge stations (EVCSs), renewable energy sources (RESs), and shunt capacitors (SCs). The actual power ranges of the EVCSs of the Vinfast company in Vietnam are used to check the stabilization of the IEEE 85-node distribution power grid by considering four penetration levels of EVCSs, namely 25%, 50%, 75%, and 100%. All penetration levels of EVCSs violate the operating load voltage limits, and the grid cannot work for all the penetration levels. Different scenarios are performed to find the minimum RES penetration level and the most possible SC penetration level to satisfy the operating voltage limits. The use of only SCs cannot satisfy the voltage limits even for the 25% EVCS penetration level. The placement of RESs provides the capability to maintain voltage within the allowed range for 25% and 50% EVCS penetration but not for 75% and 100%. Using both RESs and SCs, the operating voltage limits are satisfied by using RESs with 1385 kW (about 30.44% of loads and EVCSs) and SCs with 2640 kVAr for the 75% EVCS penetration level and using RESs with 2010 kW (about 38.58% of loads and EVCSs) and SCs with 2640 kVAr (100% of loads) for the 100% EVCS penetration level. The study indicates that the installation of EVCSs should be calculated for stable operation of the distribution power grid, and the combination of both RESs and SCs can satisfy the maximum penetration level of EVCSs in the distribution power grids.

## 1. Introduction

### 1.1. Motivation

The undesired consequences of global warming or climate change have recently become more apparent than ever [1,2]. Specifically, the world has witnessed a noticeable reduction in agricultural produce. The whole ecosystem is declining in diversity due to the overuse of fossil fuels and environmental destruction activities that serve the new industrial resolution. As a result, biodiversity losses, rising sea levels, and other natural disasters have become more frequent and of higher intensity. Moreover, other aspects directly integrated into human life, including air quality, water resources, plants, or even the economy, are highly damaged. The root of climate change and global warming is the uncontrollable increase in CO_2_ concentration, which has been exacerbated by industrialization and urbanization processes in the atmosphere [3]. The Paris Agreement, which acknowledges the prolonged adverse effects of having too much CO_2_ inside the globe, has been proposed [4]. According to the agreement, all the joined nations must commit to the process to cut CO_2_ emissions in the future. Every country will decide on specific methods and practical implementations depending on its development situation.

The transition to electric vehicles (EVs) is considered one of the most efficient and cost-effective methods to shorten CO_2_ emissions in various countries [5]. According to the research, the Australian car fleet is taken as an example, contributing 8% of the CO_2_ emission of the whole country. Two scenarios for switching to EVs have been proposed, including high-cost and low-cost ones. After a series of calculations and considerations, the authors concluded that low-cost scenarios are the better implementation for the nation. Regarding developing and popularizing EVs, China is acknowledging the lead countries [6]. According to the study, the Chinese government has planned to reach the goal of neutralizing CO_2_ before 2060, as committed to the Paris Agreement, and the EV strategy is one of the main focuses to promote the whole country toward the goal. Due to the unprecedented growth of the EV fleet, the placement of electric vehicle charging stations (EVCSs) must be precisely planned to meet the demand within all regions. Besides, policies and government support must be taken in advance to reach the rapid growth of EVs in the whole country. The authors in [7] present an overview of the aspects that highly affect the growth of electric vehicles, which are batteries, and the scale of these industries with the development of different technologies. Three of the main types of batteries, including lead-acid, hydride, and lithium-ion, are evaluated in terms of their performance and market share in the current situation of the EV industry. Additionally, the future development of these battery technologies is also given. The role of EVCSs is also emphasized in [8]. Notably, the author states that the shortening of EVCSs, which are mainly fast-charging stations, has stopped the US from popularizing EVs compared to other nations. By visualizing the current distribution status of EV fleets and the number of existing EVCSs using the available data, the authors have pointed out the largely unbalanced distribution of EVCS placement across the country. In conclusion, the authors have shown a high demand for EV infrastructure, especially the EVCSs in many areas, and also require building a transportation corridor linking all the regions to support the EV and EV market growth. In [9], the placement of EVCSs is continuously evaluated in rural areas of the United Kingdom. The author indicates that the investment of private organizations in such areas is inferior due to the rate of use, the adaptability of the current infrastructure, and, more importantly, the effectiveness of investment costs. To attract investment in EVCSs, the authors have applied the nonlinear model to estimate the payback point of time, then proposed different strategies to reduce cost by incorporating the electricity price.

### 1.2. Literature Review

As analyzed above, the importance of EVCSs is undeniable in developing a sustainable EVCS strategy. However, EVCSs are mostly integrated with distribution power systems (DEPSs) and lead to an increase in load demand value or negative impacts on the safety and reliability of DEPS operation; therefore, the placement of EVCSs in the DEPSs must be carefully planned and well optimized [10]. To partly alleviate the downsides of placing EVCSs on the DEPSs, the authors have optimized the placement of the shunt capacitors (SCs) alongside EVCSs to achieve the minimum value of power loss and net profit and also improve the reliability of the considered DEPSs [11]. The study solved the IEEE 33-node and 34-node DEPSs with the application of two optimization algorithms, including Particle Swarm Optimization (PSO) and Grey Wolf Optimization (GWO). In [12], the optimization of SCs in combination with EVCSs was also conducted. Unlike [11], the authors have executed their research in different configurations of DEPSs with the primary objective function of reducing power loss but still maintaining the voltage profile in acceptable ranges [12]. A hybrid method called Eurasian Oystercatcher Optimizer (EOO) and Quantum Neural Network (QNN) is applied to determine the optimal placement of SCs along with EVCSs. The results achieved by EOO-QNN are also compared to other methods such as WHO, SSA, and PSO, and their real effectiveness is proved through comparisons.

In [13], the authors have considered the placement of ECVSs in the 52-node DEPS using PSO with three charging output levels, namely levels 1, 2, and 3. The authors also acknowledged that the operation of those three levels of EVCS negatively affected the stable operation of the considered grid, especially level 3. The authors then proposed placing the photovoltaic generators (PVs) on the grid beside EVCSs to reduce cost, power loss, and transformer loading and, thus, ensure the regular operation of the given grid. In [14,15], the placement of PVs combined with EVCSs was also conducted in the same configuration of DEPSs, which was the IEEE-33 node, to achieve the minimum power loss value and maintain the other important parameter of the given grid. However, the authors in [14,15] focused on the proposed method’s real effectiveness. Particularly, an improved version of the Jaya Algorithm (JAYA) was applied [14], and the results achieved by the JAYA have proven it was superior to other methods. In [15], the Balanced Mayfly Algorithm (BMA) was implemented, and its capability was demonstrated when compared to others, such as GAIPSO and the original version of the Mayfly Algorithm (MA).

In [16], renewable energies (REs) and battery energy systems (BESs) were optimally allocated to the IEEE 33-node and the 136-node Brazil DEPS to reduce the adverse effects caused by EVCS load. The paper evaluated and analyzed the variation of base load demand, RES uncertainties, and EVCS load within 96 h. Chaotic student psychology-based optimization (CSPBO) was executed to determine the optimal location of photovoltaic generator-based BESs (SG-BESs) and wind generator-based BESs (WT-BESs). The results obtained by CSPBO indicated that the method is more effective than the original version, student psychology-based optimization (SPBO), regarding the ability to avoid local optimal and convergence speed while being applied to solve the problem. The authors in [17] have combined a distribution static compensator (DSTATCOM), battery energy storage system (BESS), and RES to deal with the unbalanced effects posted by EVCS to both IEEE 33- and 69-nodes. The Slime Mold Algorithm (SMA) is implemented to determine the best allocation of DSTATCOMs, BESSs, and REs for power loss minimization. Besides, voltage profile and system reliability must be ensured while solving the considered problem. Moreover, the results achieved by the SMA have proven its superiority to other methods, such as the Cuckoo Search Algorithm (CSA), Bat Algorithm (BA), and African Vulture Optimization Algorithm (AVOA).

In [18], the authors focused on solving the power loss and voltage fluctuation in the DEPS while having the presence of EVCSs. The author then proposed the integration of DSTATCOMs to the 28- and 108-node DEPSs for power loss reduction and minimizing the voltage stability index (VSI) and annual net savings cost. A novel meta-heuristic algorithm called Bald Eagle search algorithms (BESA) is applied to optimize the allocation of those DSTATCOMs placed on the grid to reach the desired value of the considered objective functions. The results obtained by BESA are superior to other methods, such as the Bat algorithm (BA), the African Vulture Optimization Algorithm (AVOA), the Binary Bat Algorithm (BBA), and Particle Swarm Optimization (PSO). Similar to [18], loss reduction and voltage improvement are also the main concerns of the authors in [19] while integrating EVCSs into the 83-node practical DEPS in Taiwan. However, the authors in [19] combined DGs and SCs to cope with those concerns using a meta-heuristic algorithm called the Marine Predator Algorithm (MPA). Moreover, DSTATCOMs were also combined with DGs in [20] to not only reach the minimal value of active power loss and voltage node but also provide the capability of correct power factor, which plays a vital role in maintaining the effectiveness of grid operation and the improve the efficiency of electrical devices integrated with the grid. In the research, a fuzzy-based optimization method is implemented to optimize the placement of DSTATCOMs and DGs in the IEEE 69-node to reach the goals mentioned.

Besides power loss reduction and voltage stability, which are almost the two main concerns while integrating EVCSs into a given DEPS, as mentioned in the studies above, other focuses are also taken into account, such as minimizing investment cost and annual energy loss cost with different load factors [21], minimizing the investment and operation cost of EVCSs [22], and maximizing power supply profit [23].

Instead of solving the particular problem while integrating EVCSs into different configurations of DEPSs, other research is concentrated on presenting a detailed look at the state development of EVs and the related infrastructure to serve the growth of EVs. Notably, the authors in [24] provide an overview and evaluate different strategies while integrating the EV’s infrastructure into the large-scale DEPS. Specifically, the so-called vehicle-to-grid (V2G) approach is discussed in different ways, aiming to reach a higher degree of resilience and sustainability in the grid operation. In [25], the authors focus on clarifying the role of the auxiliary services to serve the V2G approach and urge for the modernization of the DEPS to adapt to the higher demand for EVs. Other challenges in terms of engineering, economic development, and government policies are also addressed. In [26], the authors mainly discussed the technical obstacles and the standardization process of EVCS in India. Various types of EVCSs, charging modes, connectors, and charging levels are mentioned in detail. The study highly encouraged the implementation of the combined charging system (CSS), which provides the ability to support both AC and DC charging. In [27], the research focused on discussing and evaluating the situation where home charging stations (HCSs) are becoming more popular due to the increase in EV owners. As a result, the load demand in the power grid is also more significant, and renewable energy sources (RESs) are affordable solutions for electric companies to meet the extra load demanded by HCSs. Next, policies, encouragement, and government support regarding the development of EV infrastructure are given. Moreover, the technical challenges of using RESs combined with EVCSs in some residential areas in Germany were also evaluated.

In this research, EVCSs, capacitors, and renewable power sources are considered to optimize their placement in the IEEE 85-node DEPS to achieve different objective functions as follows:

(1) Minimizing the overall active power loss;
(2) Minimizing the total capacity of all renewable power sources;
(3) Minimizing the total voltage deviation index.

Two meta-heuristic algorithms, namely the Chameleon Swarm Algorithm (CSA) [28] and Snow Geese Algorithm (SGA) [29], are applied to determine the optimal placement of EVCSs and other auxiliary devices on the grid to reach the minimum value of the three objective functions in different scenarios. The main novelties can be listed as follows:

- Apply newly published meta-heuristic algorithms, which were recently published: the purpose is to find the most suitable algorithm for the problem;
- Classify different zones in distribution electric power systems to install EVCSs: A group of distribution lines is selected to install several levels of EVCSs. The purpose is not to install many EVCSs close together;
- Implement different penetration levels of EVCSs, with 25%, 50%, 75%, and 100%, in distribution electric power systems: the purpose is to survey the operating limits of the grids to avoid low voltage for base loads;
- Implement the installation of capacitors in the distribution electric power systems corresponding to the different levels of EVCSs: the purpose is to enhance the operation limits of the grids with a low investment cost for installing capacitors;
- Implement the installation of photovoltaic systems in distribution electric power systems corresponding to the different levels of EVCSs: the purpose is to enhance the operation limits of the grids with a higher investment cost for installing photovoltaic systems than capacitors;
- Combine the simultaneous placement of capacitors and photovoltaic systems in distribution electric power systems: the use of both reactive and active power sources can improve voltage and reduce energy loss.

After running the algorithms for several simulation scenarios in the IEEE 85-node distribution electric power system, the study’s contributions are as follows:

- Determining the most effective meta-heuristic algorithm to find the optimal solution for each case study considered across all four scenarios implemented in the research;
- Selecting the best penetration level of EVCSs in distribution electric power systems that can support electric vehicles highly and stabilize the operation of the distribution of electric power systems;
- Selecting the most suitable power of capacitors for each penetration level of EVCSs: If capacitors can compensate for the voltage drop, their most optimal capacity is selected. In some cases, with very high penetration levels of EVCSs, capacitors cannot compensate for enough reactive power, and the grid is warned not to install more EVCSs;
- Selecting the most suitable power of photovoltaic systems for each penetration level of EVCSs: For all cases with very high penetration levels of EVCSs, the system can compensate enough active power for the grid to still work stably and effectively; however, the high power of the systems can lead to high investment costs;
- Using the simultaneous placement of capacitors and photovoltaic systems is very good for keeping voltage in a stable operation range, and the cost is more suitable than the use of only photovoltaic systems.

Besides the introduction, the other content of the research is structured as follows: Section 2 will present the main objective functions and the involved constraints; Section 3 briefly describes the applied methods focused on their distinctive features; Section 4 presents the results and related detailed analysis regarding the actual effectiveness of the applied methods and different employments of the considered problem; and lastly, Section 5 will reveal the essential conclusions of the whole research, including the downsides and the future orientation of the research.

## 2. Problem Description

**Figure 1:**
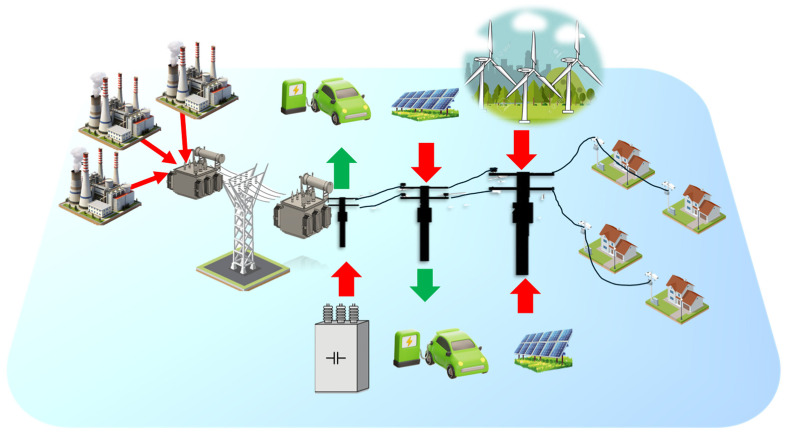
A power system with EVCSs, renewable energy, and capacitors.

This research considered three different objectives functions while optimizing the allocation of EVCSs in different penetration levels to the DEPS, including (1) minimizing the total active power loss (TPWL), (2) minimizing the total voltage deviation index (TVDI), and (3) minimizing the total rated power of photovoltaic systems ($P_{allPV}$). Figure 1 below illustrates the power system where the DEPS is integrated with EVCSs and different auxiliary devices. Additionally, the mathematical expression of the three objective functions and all the related constraints will be given in the next subsections.

### 2.1. The Objective Function Considered

The first objective function: This objective function concentrates on reducing the total active power loss (TPWL) during the power distribution process on all lines of the given DEPS. The objective is determined as follows.

(1) $$Reduce\;TPWL=\sum_{dl=1}^{{No}_{dl}}\left(3.{Ic}_{dl}^{2}.{Re}_{dl}\right)$$

where TPWL is the overall active power loss on the given distribution electric power system (DEPS); ${Ic}_{dl}$ is the current amplitude on the branch *dl* of the given DEPS; ${Re}_{dl}$ is the resistance of the branch *dl*; and ${No}_{dl}$ is the number of distribution lines.

The second objective function: This second objective function is to minimize the capacity of all added PV systems in the grid so that the voltage of all nodes is within the operating range (it is supposed to be between 0.95 and 1.05 pu). The second objective function is formulated as follows:

(2) $$Minimize\;P_{allPV}=\sum_{y=1}^{{No}_{pv}}P_{pv,y}$$

where $P_{allPV}$ is the total capacity of all installed PV systems in the grid; $P_{pv,j}$ is the capacity of the *yth* installed PV system; and ${No}_{pv}$ is the number of installed PV systems.

The third objective function: This third objective function is mainly about minimizing the total voltage deviation index (TVDI) at all nodes in the whole considered DEPS, which is formulated by the following expression.

(3) $$Minimize\;TVDI=\sum_{n=1}^{{No}_{n}}\left|1-V_{n}\right|$$

where $V_{n}$ is the voltage magnitude at node *n*; ${No}_{n}$ is the number of nodes in the grid.

### 2.2. The Constraint Involved

- Power balance constraints: These constraints focus on ensuring the dynamic balance of the DEPS, which is represented by the correspondence between the total supplied power and the total demanded power with the loss amount. The constraints are applied for both active and reactive power, as shown by the following expressions:

(4) $$P_{grid}+\sum_{y=1}^{{No}_{pv}}P_{pv,y}=\sum_{n=1}^{{No}_{n}}P_{Load,n}+{No}_{LV1}.P_{LV1}+{No}_{LV2}.P_{LV2}+{No}_{LV3}.P_{LV3}+TPWL$$

(5) $$Q_{grid}+\sum_{c=1}^{{No}_{cb}}Q_{cb,c}=\sum_{n=1}^{{No}_{n}}Q_{Load,n}+TRPL\;$$

where TRPL is the total reactive power loss in the grid obtained by

(6) $$TRPL=\sum_{dl=1}^{{No}_{dl}}\left(3.{Ic}_{dl}^{2}.{Reac}_{dl}\right)$$

- Operational voltage constraint: Voltage is a crucial factor that directly affects the operational status of all the electrical devices connected to the grid. Therefore, the voltage must be maintained in an allowed range between the minimum and maximum values to ensure the reliability and stability of a particular DEPS [30,31]. The constraint must meet the inequality below:

(7) $$V^{min}\;\leq \;V_{n}\;\leq V^{max};n=1,\;\ldots ,\;N_{nd}$$

where $V^{min}$ and $V^{max}$ are the minimum and maximum allowable voltage limitations.

- Thermal constraint: This constraint mainly concerns the current amplitude circulated through a particular branch, which must be lower than the allowed value. Unlike voltage, the current amplitude needs to respect the maximum value only, as given in the expression below:

(8) $${Ic}_{dl}\;\leq {\;Ic}_{dl}^{max}$$

where ${\;Ic}_{dl}^{max}$ is the maximum current amplitude allowed to circulate through the *dlth* branch.

- Operating constraint of installed PV systems: This constraint means that PVs can inject a particular amount of active power into the grid within their minimum and maximum limits as described by the following equation:

(9) $$P_{pv}^{min}\;\leq \;P_{pv,y}\;\leq P_{pv}^{max}$$

where $P_{pv}^{min}$ and $P_{pv}^{max}$ are the minimum and maximum capacity limits of all installed PV systems.

- SC’s operational constraints: Similar to PV systems, the amount of reactive power supplied by SCs can only vary in an interval between the minimum and maximum bounds as follows:
  (10) $$Q_{cb}^{min}\;\leq \;Q_{cb,c}\;\leq Q_{cb}^{max}$$
  In addition, the total reactive power supplied by all capacitor banks must be lower or equal to the demands of loads and loss in lines, as expressed in the following inequality:
  (11) $$\sum_{c=1}^{{No}_{cb}}Q_{cb,c}\leq \sum_{n=1}^{{No}_{n}}Q_{Load,n}+TRPL$$

- Location constraints of PV systems: This constraint indicates that the placements of installed PV systems must satisfy the geography constraint. In the study, PV systems can be placed wherever, excluding the slack node, as shown in the following model:

(12) $$2\;\leq \;{Lo}_{pv,y}\;\leq {No}_{n}$$

- Location constraints of EVCSs: The study supposes that EVCSs are limited to the installed locations. This means that the EVCSs can be found in some feeders and not in others. The assumption is due to the difference of residences on different roads. Typically, EVCSs are really necessary for centers and not for rural areas. Thus, the locations of EVCSs are constrained by the following model:

(13) $${Lo}_{LV1}\in {SLo}_{LV1}$$

(14) $${Lo}_{LV2}\in {SLo}_{LV2}$$

(15) $${Lo}_{LV3}\in {SLo}_{LV3}$$

## 3. The Applied Algorithm

### 3.1. Chameleon Swarm Algorithm

In this subsection, the main steps of the whole optimizing process of CSA will be presented along with a specific mathematical model. Particularly, five main steps are sequentially executed while applying CSA to an optimization problem: the initialization, the evaluation, the update process, the boundary check, and the refining procedure.

#### 3.1.1. The Initialization

In this step, a set of random individuals will be produced using the following model:

(16) $$S_{n}=S_{n}^{min}+rnd\times \left(S_{n}^{max}-S_{n}^{min}\right)$$

where $S_{n}$ is the individual *n* of the population with *n* = 1, 2, …, *N_Ps_* with *N_Ps_* is the population size; rnd is a random value between zero and one; $S_{n}^{max}$ and $S_{n}^{min}$ are the maximum and minimum solutions of the search space.

#### 3.1.2. The Evaluation

This step is executed to evaluate the quality of each individual (solution) in the population at the time while the new solutions are produced. The mathematical expression of this step is given as follows:

(17) $${FN}_{n}=OJF(S_{n})\;with\;n=1,\;2,...,\;N_{Ps}$$

where ${FN}_{n}$ is the fitness value of the individual *n*; OJF the main objective function featured by the considered optimization problem.

#### 3.1.3. The Update Process

According to [28], the update process comprises two main stages: Stage 1—seeking and detecting, and Stage 2—maneuvering and attacking. Note that these two stages are subsequently executed in the whole search process. The mathematical mode for each stage will be given as follows

- *Stage 1: Seeking and detecting.*

In this first phase, all the individuals are free to move within the search space to seek potential prey. The location of each individual in Stage 1 is given as the following expression:

(18) $$S_{n}^{new\_s1}=\left\{\begin{array}{c} S_{n}+{ef}_{1}\times \left(S_{PB,n}-S_{GB}\right)\times \omega_{1}+{ef}_{2}\times \left(S_{GB}-S_{n}\right)\times \omega_{2}\;\;\;\;\;if\;rf\;\geq 0.1\\ S_{n}+\varepsilon \times \left({ub}_{n}-{lb}_{n}\right)\times \omega_{3}\left(rand-0.5\right)\;\;\;\;\;\;\;\;\;\;\;\;\;\;\;\;\;\;\;\;\;\;\;\;\;\;\;\;\;\;\;\;\;\;\;\;\;\;\;\;\;\;\;\;else \end{array}\right.$$

where ε is an adaptive factor obtained by:

(19) $$\varepsilon ={\delta \times e}^{{\left(-\frac{\alpha \times {Ir}_{h}}{{Ir}_{H}}\right)}^{\beta }}$$

- *Stage 2: Maneuvering and attacking.*

Each individual executes two practices in this stage, maneuvering and attacking, before capturing the prey. Particularly, the individual must adjust its velocity toward the prey and, after that, quickly reach the location of the prey. Those two practices are simulated using the following mathematical models:

(20) $$V_{n}^{new}=IW\times V_{n}+{IF}_{1}\times \left(S_{GB}-S_{n}\right)\times \omega_{4}+{IF}_{2}\times \left(S_{PB,n}-S_{n}\right)\times \omega_{5}$$

(21) $$S_{n}^{new\_s2}=S_{n}+\frac{{\left(V_{n}^{new}\right)}^{2}-{\left(V_{n}\right)}^{2}}{2\times \mu }$$

where IW and μ are the inertia and accelerating factors, which are detemined by

(22) $$IW={\left(1-\frac{{Ir}_{h}}{{Ir}_{H}}\right)}^{\left(\sqrt[{ep}]{\frac{{Ir}_{h}}{{Ir}_{H}}}\right)}$$

(23) $$\mu =2590\times \left(1-e^{-log({Ir}_{h})}\right)$$

#### 3.1.4. The Boundary Check

This step is applied to ensure that all the new solutions are legal and located inside the search space that initially featured the considered problem. The legal solutions are kept unchanged and can be joined to the next step; otherwise, the new solutions will be replaced by the maximum or the minimum solution for the case of violating the upper and the lower boundaries. The mathematical expression of this step is given as follows:

(24) $$S_{n}=\left\{\begin{array}{c} S_{n}^{min}\;\;\;\;\;\;\;\;\;\;\;\;\;\;\;\;\;\;\;\;\;\;\;\;if\;\;S_{n}<S_{n}^{min}\\ S_{n}\;\;\;\;\;\;\;\;\;\;\;\;\;if\;S_{n}^{min}\leq \;S_{n}\leq S_{n}^{max}\\ S_{n}^{max}\;\;\;\;\;\;\;\;\;\;\;\;\;\;\;\;\;\;\;\;\;\;\;\;\;if\;S_{n}>S_{n}^{max} \end{array}\right.$$

#### 3.1.5. The Refining Procedure

This procedure is implemented anytime when new solutions are newly produced to retain the high-quality solutions for the next iteration and eliminate the lower ones by considering the fitness value of each solution:

(25) $${FN}_{n}=\left\{\begin{array}{c} {FN}_{n}^{new},\;\;\;\;\;\;\;\;if\;{FN}_{n}^{new}<{FN}_{n}\\ {FN}_{n},\;\;\;\;\;\;\;\;\;\;\;\;\;\;\;\;\;\;\;\;\;\;\;\;otherwise \end{array}\right.\;$$

(26) $$S_{n}=\left\{\begin{array}{c} S_{n}^{new},\;\;\;\;\;\;\;\;if\;{FN}_{n}^{new}<{FN}_{n}\\ S_{n},\;\;\;\;\;\;\;\;\;\;\;\;\;\;\;\;\;\;\;\;\;\;\;\;otherwise \end{array}\right.$$

where ${FN}_{n}^{new}$ is the fitness value of the new solution $S_{n}^{new}$ after the update process mentioned in Section 3.1.3.

**Figure 2:**
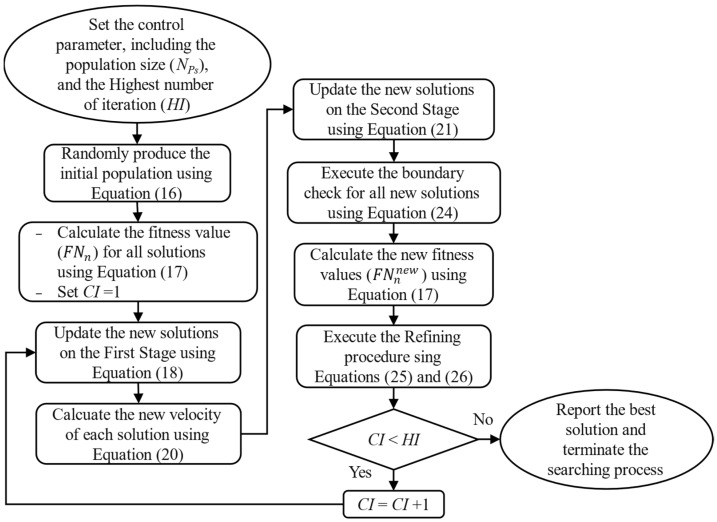
The whole searching process of CSA.

The whole searching process of CSA while dealing with optimization problems is shown in Figure 2 below:

### 3.2. Snow Geese Algorithm

Snow geese are renowned for their migratory behavior [29], traveling to locations with more favorable living conditions during specific times of the year. Throughout their migration, these birds commonly employ two flight formations: the herringbone and the straight line. These flight patterns are observed in various bird species that undertake migrations. The herringbone formation is particularly advantageous due to its aerodynamic efficiency. Birds flying behind benefit from the upward airflow generated by the wings of the birds in front, reducing their energy expenditure. This formation also minimizes air resistance. Furthermore, it enhances visual communication and coordination within the flock, aiding in navigation and maintaining group cohesion.

Being a meta-heuristic algorithm, the optimizing process of SGA also implements the main steps similar to CSA described in Section 3.1. The main difference between SGA and CSA is the update process for new solutions. Hence, this section only focuses on describing and modeling the two stages of the update process; the other steps of the optimizing process remain the same, as seen in Section 3.1.

The simulation of these two flight formations serves as the foundation for developing an updated method for SGA’s new solutions.

- *Stage 1: The herringbone method*

In this first stage, all the individuals of the snow geese flock apply the herringbone flying method. The mathematical expression of this flying method is simulated as follows:

(27) $$S_{n}^{new\_s1}=\left\{\begin{array}{c} S_{n}+V_{n}\times {AF}_{1}\times \left(S_{GB}-S_{n}\right)+V_{n}^{new},\;\;\;\;\;\;\;\;\;\;\;\;\;\;\;\;\;\;\;\;\;\;\;\;\;\;\;\;\;\;\;\;\;\;\;\;\;\;\;\;\;\;\;\;\;\;\;\;\;\;\;\;\;\;\;\;\;\;\;\;\;\;\;\;\;\;\;\;\;\;\;\;\;\;\;\;\;\;\;\;\;\;\;\;if\;n\;\leq \frac{N_{Ps}}{5}\\ S_{n}+V_{n}\times {AF}_{1}\times \left(S_{GB}-S_{n}\right)+{AF}_{2}\times \left(S_{CT}-S_{n}\right)+V_{n}^{new},\;\;\;\;\;\;\;\;\;\;\;\;\;\;\;\;\;\;\;\;\;\;\;\;\;\;\;\;\;\;\;\;\;\;\;\;\;if\;\frac{N_{Ps}}{5}\leq n\leq \frac{{4N}_{Ps}}{5}\\ S_{n}+V_{n}\times {AF}_{1}\times \left(S_{GB}-S_{n}\right)+{AF}_{2}\times \left(S_{CT}-S_{n}\right)+{AF}_{3}\times \left(S_{WS}-S_{n}\right)+V_{n}^{new},\;\;\;\;\;\;\;\;\;\;\;\;\;\;\;\;\;otherwise \end{array}\right.$$

where the new velocity $V_{n}^{new}$ is obtained by using Equation (28), and $S_{CT}$ is the central individual determined by using Equation (29).

(28) $$V_{n}^{new}={\mu \times V}_{n}+Rf$$

(29) $$S_{CT}=\frac{\sum_{n=1}^{N_{Ps}}S_{n}\times {Ft}_{n}}{N_{Ps}\times \sum_{n=1}^{N_{Ps}}{Ft}_{n}}$$

- *Stage 2: The straight-line method*

In this stage, the flying method is simulated using the following expression:

(30) $$S_{n}^{new\_s2}=\left\{\begin{array}{c} S_{n}+\left(S_{n}\times S_{GB}\right)\times \omega_{6},\;\;\;\;\;\;\;\;\;\;\;\;\;\;\;\;\;\;\;\;if\;\omega_{6}>0.5\;\\ S_{n}+\left(S_{n}\times S_{GB}\right)\times \omega_{6}\times BR,\;\;\;\;\;\;\;\;else \end{array}\right.$$

The optimizing process of SGA does not execute all the update methods for the new solution as mentioned in Stage 1 and Stage 2; consequently, the update can be executed using one of two update methods, as mentioned through the determination of a factor called the selection term, which is calculated as follows:

(31) $$st=\frac{2\times \pi \times CI}{HI}$$

where *CI* and *HI* are the current and the highest index of iteration; π is approximately set at 3.1416.

After st is determined, the mechanism for selecting the specific update method is determined as follows:
If *st* < π Executing the update process mentioned in Stage 1, Otherwise
Executing the update process mentioned in Stage 2, End. 

**Figure 3:**
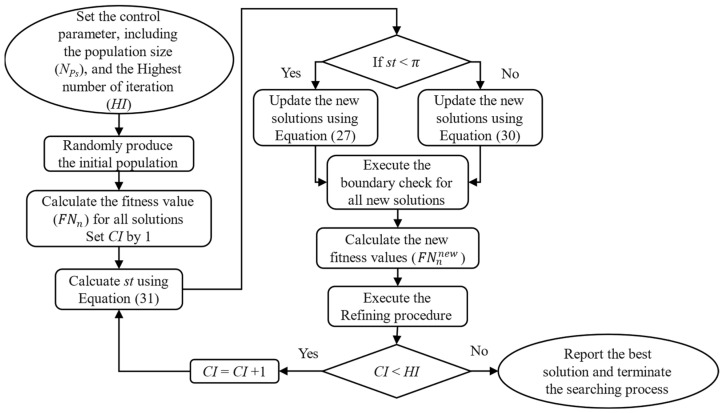
The whole searching process of SGA.

The whole searching process of SGA while dealing with optimization problems is shown in Figure 3 below:

## 4. Results

In this section, CSA and SGA are applied to optimize the allocation of EVCSs. All study cases are given in Table 1. For each case, CSA and SGA are tested by 50 trial runs for the best solution before any comparison of their performance. In addition, penetration levels of EVCSs and other information on added electric components are given in Table 2 and Table 3.

**Figure 4:**
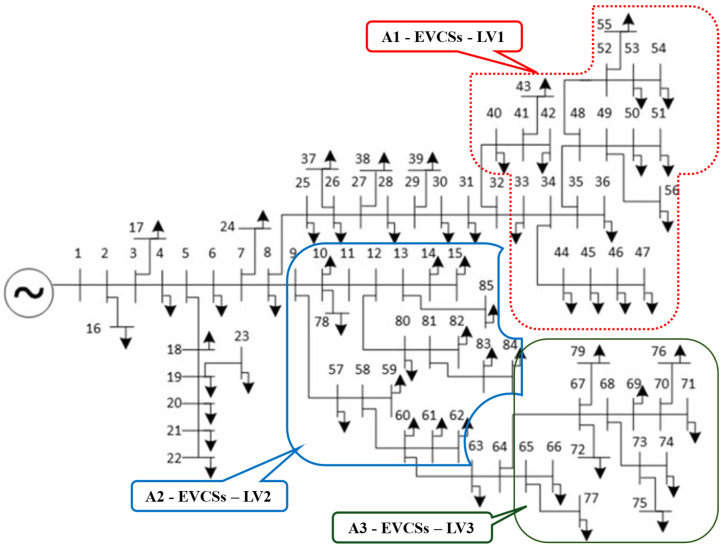
The restriction on legal positions of EVCSs for each level.

On the other hand, the placement of EVCSs in the distribution electric power system, in practice, must consider the geographical aspects. The EVCSs are often placed near residential areas, malls, or city centers to offer high reachability to EV owners and other involved services. Considering this aspect, suppose that the EVCSs and each level are only placed in specific areas, as shown in Figure 4. This study supposes that the locations for EVCS placement are restricted, and Figure 4 shows the IEEE 85-node DEPS with possible suggested locations. The detailed specifications of the selected DEPS are cited from [33].

All the works of this study are conducted on a personal computer with the basic comparison as follows: a 2.6 GHz central processing unit (CPU) paired with 16 GB of random accessing memory (RAM). MATLAB programming language version R2019a is the main foundation for performing all coding and related simulations.

### 4.1. The Results Obtained in Scenario 1

**Figure 5:**
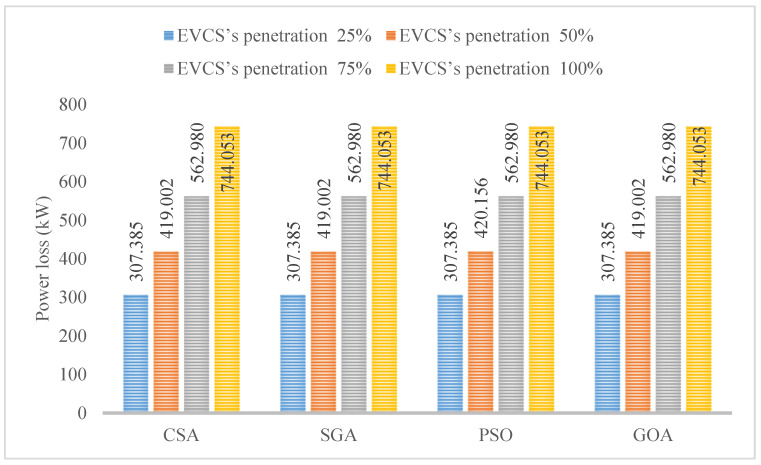
The minimum total active power loss obtained by the two applied algorithms in the first scenario.

The results obtained by the two applied methods are shown in Figure 5. The TPWL achieved by CSA and SGA is the same at all four penetration levels. Besides, two other meta-heuristic algorithms, including one that was recently published, called the Golf Optimization Algorithm (GOA) [34], and Particle Swarm Optimization (PSO) [35] are also executed for side-by-side comparison. It is very clear that the value of TPWL became more prominent while the penetration of EVCSs increased, particularly from 307.385 kW to 744.053 kW for both algorithms, corresponding to the lowest and the highest penetration levels. The optimal locations of EVCSs of each level are reported in Table A1 of the Appendix A.

**Figure 6:**
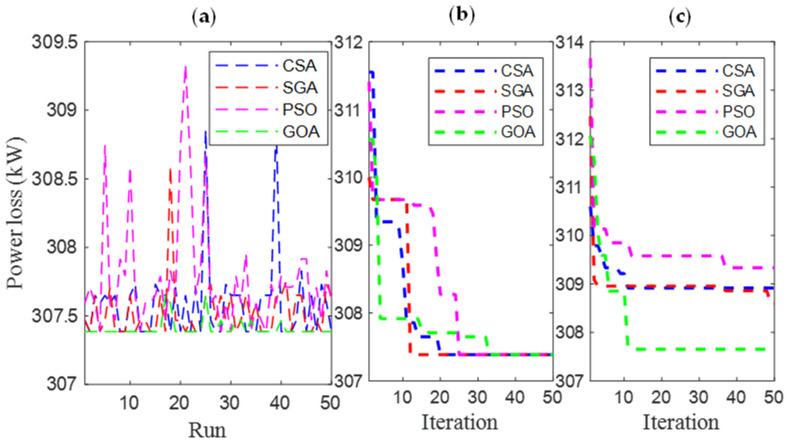
The results of (**a**) 50 test runs; (**b**) the minimum convergences; and (**c**) the maximum convergence obtained by CSA and SGA for 25% penetration of EVCSs in Scenario 1.

**Figure 7:**
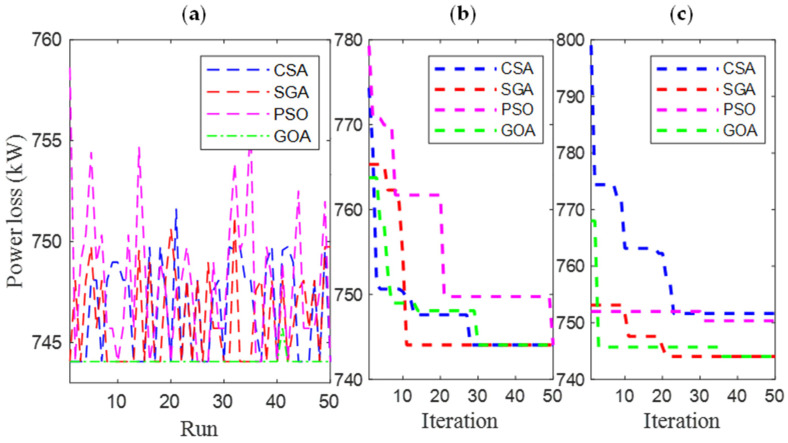
The results of (**a**) 50 test runs; (**b**) the minimum convergences; and (**c**) the maximum convergence obtained by CSA and SGA for 100% penetration of EVCSs in Scenario 1.

Figure 6 and Figure 7 present the results obtained by CSA, SGA, PSO, and GOA across various criteria, corresponding to two different EVCS penetration levels, 25% and 100%. These include power loss values after 50 test runs (Figure 6a and Figure 7a), minimum convergence (Figure 6b and Figure 7b), and maximum convergence (Figure 6c and Figure 7c). Figure 6a and Figure 7a show that GOA is the most stable algorithm among the four algorithms, achieving the lowest fluctuation among 50 test runs. In this regard, the stability of SGA and CSA is ranked second and third, respectively, and finally, PSO is the most unstable algorithm. Although GOA is the most stable algorithm, GOA has failed to maintain its effectiveness compared to CSA and SGA, considering the minimum convergence speed, which is present in Figure 6b and Figure 7b, respectively. These two subfigures provide minimum convergence curves for both algorithms at the two EVCS penetration levels. Observations from these figures show that, although both CSA and SGA reach the same minimum power loss value at the final iteration, SGA offers a faster convergence speed than CSA at both penetration levels. Specifically, for the 25% EVCS penetration case (Figure 6b), SGA requires approximately 25 iterations to reach the optimal power loss value, while CSA requires over 30. For the 100% EVCS penetration case, SGA’s superiority is more pronounced, reaching the minimum power loss in only 10 iterations, compared to over 30 for CSA. PSO specifically shows its faster convergence speed compared to GOA for the 25% EVCS penetration but lags behind GOA for the 100% EVCS penetration. Finally, Figure 6c and Figure 7c display the maximum convergence curves achieved by four algorithms. It is evident that SGA is superior to CSA in this aspect, demonstrating both a faster convergence speed and better maximum power loss values. However, GOA is the algorithm that achieves the best maximum convergence speed compared to the remaining algorithms.

**Figure 8:**
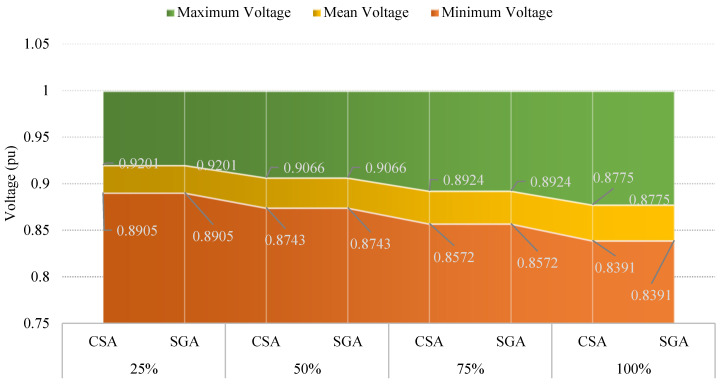
The voltage profile at all nodes obtained by the two applied methods for each level of EVCS penetration.

Figure 8 indicates that the minimum, mean, and maximum voltage values obtained by CSA and SGA are identical. However, a clear trend emerges: as the penetration level of EVCSs increases, the minimum voltage value consistently decreases. As we know, voltage plays a crucial role in determining the stability and effectiveness of most electrical devices. By acknowledging the role of voltage, the following sections will implement different solutions to improve the voltage values.

### 4.2. The Results Obtained in the Second Scenario

#### 4.2.1. The Results Obtained in Case 1

**Figure 9:**
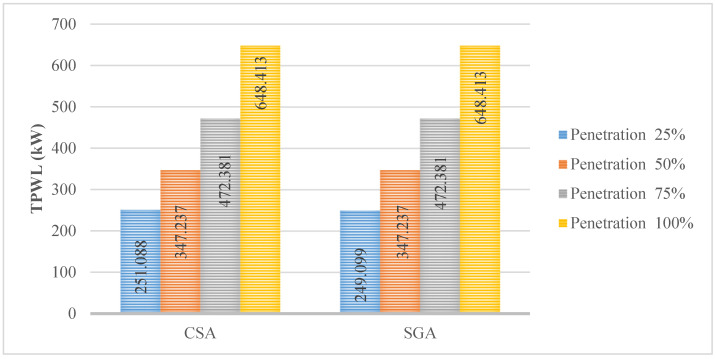
The minimum TPWL achieved by the two applied algorithms.

In this subsection, shunt capacitors (SCs) are optimized for placement beside the EVCSs, which are already allocated in the first scenario. The TPWL values achieved by CSA and SGA in this first case are shown in Figure 9. Generally, the value of TPWL at each penetration level has been significantly reduced compared to those in the first scenario. Besides, the results achieved by the two algorithms are mostly the same except for the first penetration level. In particular, the TPWL obtained by SGA in the first penetration level is only 249.099 kW, while that of CSA is 251.088 kW. The optimal locations and rated powers of SCs in this case are given in Table A2 in the Appendix A.

**Figure 10:**
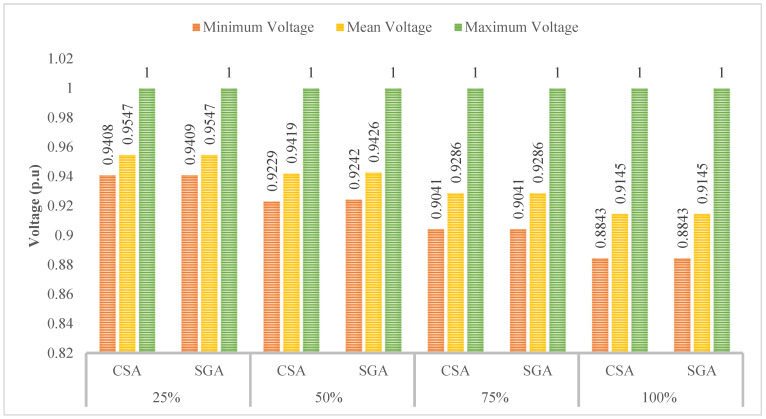
The summary of voltage values obtained by the two applied algorithms for each level of EVCS penetration.

Figure 10 shows the voltage values in terms of three different criteria, including the minimum, mean, and maximum voltage. At first glance, the minimum voltage values show a clear improvement compared to those displayed in Figure 8 of the first scenario; however, compared to the voltage limits which are mentioned in [30,31], all the voltage values reported in this scenario are still far from the point which acknowledges that all the electrical devices will work safely and efficiently, especially at the 100% penetration level of EVCSs.

#### 4.2.2. The Results Obtained in Case 2

**Figure 11:**
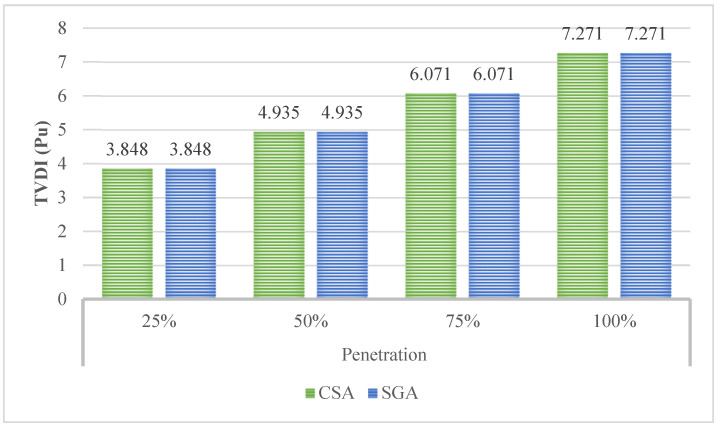
The TVDI values obtained by two algorithms in Case 2.

The results obtained by the two algorithms corresponding to each penetration level are given in Figure 11. Regarding the performance of the two algorithms, again, there is no difference between these algorithms across all four penetration levels of EVCSs. The optimal locations and rated powers of SCs in this case are given in Table A3 in the Appendix A.

**Figure 12:**
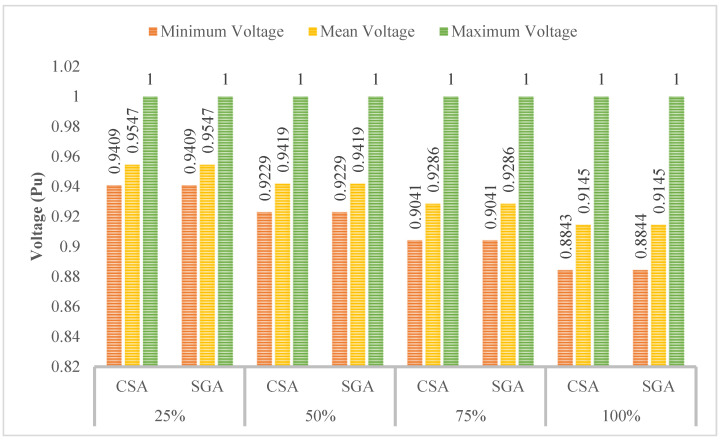
The summary of voltage values obtained by the two applied algorithms for each level of EVCS’s penetration in Case 2.

Figure 12 indicates that the minimum voltage values in all those cases are below the standard of 0.95 pu. Moreover, the more EVCS penetrates the grid, the lower the minimum voltage is.

### 4.3. The Results Obtained in the Third Scenario

#### 4.3.1. The Results Obtained in Case 1

**Figure 13:**
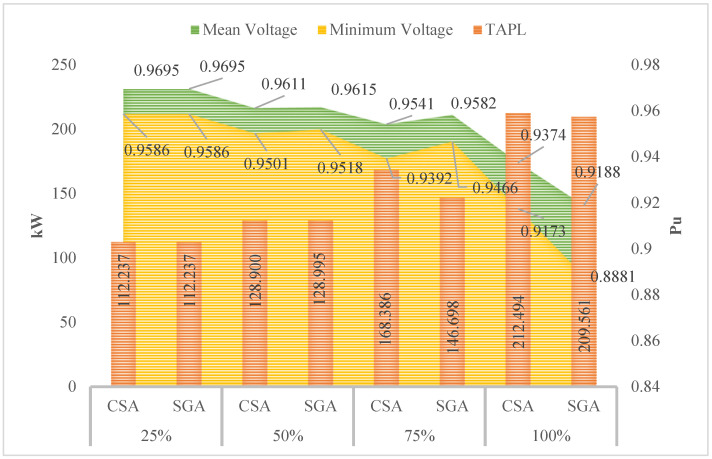
The results obtained by the two algorithms in Case 1 of Scenario 3.

The results in this case are shown in Figure 13 alongside the voltage values. Generally, the presence of PVs in the grid offers a higher degree of TPWL reduction in all four penetration levels of EVCSs compared to Case 1 in the second scenario. The optimal solution for this case is given in Table A4 of the Appendix A.

**Figure 14:**
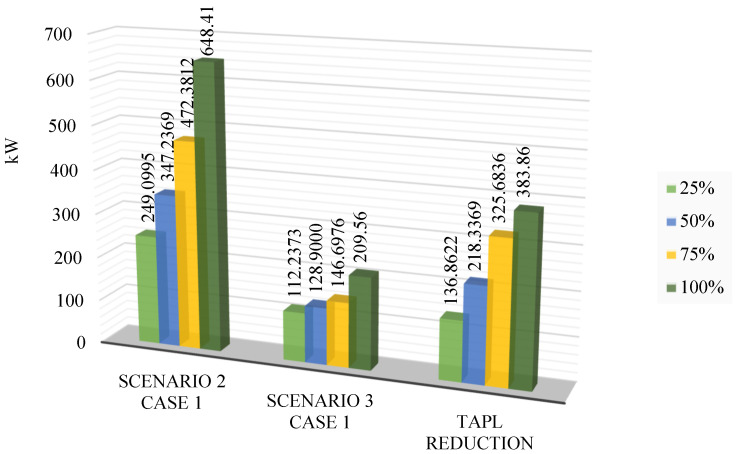
The comparison on TVDI between Case 1 of Scenarios 2 and 3.

Figure 14 shows the TPWL values obtained in Scenarios 2 and 3, focusing on minimizing TPWL for four EVCS penetration levels. The primary distinction between these scenarios is the auxiliary devices used for TPWL reduction: Scenario 2 employs SCs, while Scenario 3 utilizes PVs. Clearly, PV placement offers a significant advantage in TPWL reduction compared to SCs. This can be attributed to the operating principles of these devices. PVs directly inject real power into the grid, which powers various loads. Furthermore, real power (alternating current) is the primary input for conversion to direct current, which powers electric motors in most electric vehicles. PV connection at specific nodes reduces the power transmitted from distant main sources. Reduced power transmission translates to lower current flow through distribution lines, thereby decreasing power loss. SCs, conversely, supply reactive power, improving the voltage profile at the SC node and nearby nodes. However, SCs do not provide real power, which is essential for most loads, including electric vehicles. Consequently, when EVCSs are installed, the main power source must compensate for the real power deficit. As mentioned, this source is often located far from the EVCS position, resulting in transmission power losses proportional to the distance. Therefore, the effectiveness of SC placement is limited, particularly within the context of this research.

**Figure 15:**
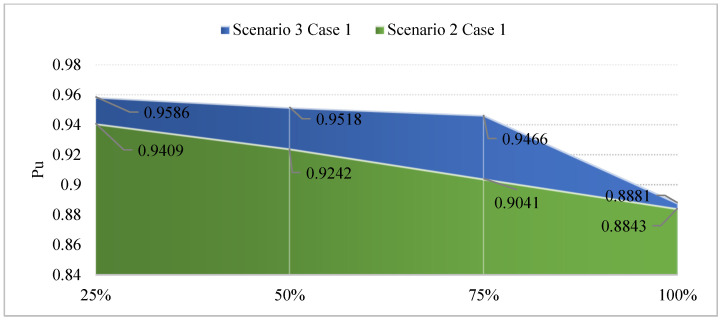
The improvement in voltage values of Case 1 in Scenario 3 compared to Case 1 of Scenario 2.

Figure 15 provides a detailed look at the voltage profile resulting from four penetration levels of EVCS in Scenario 2 and 3, with the main objective function of minimizing the TPWL value. In this figure, the advantage of SCs compared to PVs is clearly shown. In particular, the placement of SCs results in a better voltage profile over PVs in all four levels of EVCS penetration. As mentioned earlier, SCs do not supply real power but inject reactive power into the grid. The injection of reactive power provides the ability to improve the voltage profile overall, which PVs cannot achieve similarly. However, when the EVCS penetration increases, the improvement driven by reactive power supplied by SCs also decreases.

#### 4.3.2. The Results Obtained in Case 2

**Figure 16:**
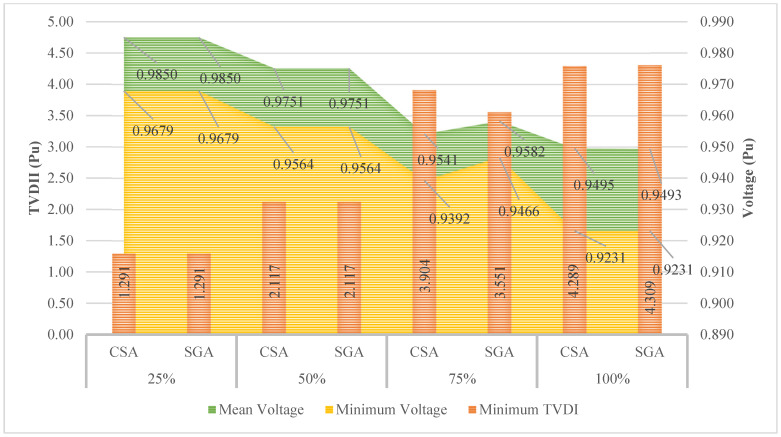
The results obtained by the two algorithms in Case 2 of Scenario 3.

Figure 16 with three elements shows the TVDI, minimum voltage, and mean voltage. In the figure, CSA and SGA result in the same value of minimum TVDI for the first two penetration levels of EVCSs, which are 25 and 50%, respectively. The differences between the minimum TVDI values obtained by the two algorithms on the last two penetration levels start to become apparent. SGA shows superiority compared to CSA on the third penetration level while reaching a better minimum value of TVDI; however, SGA lags behind CSA compared to its TVDI value achieved in the last penetration level. The optimal solution for this case is given in Table A5 of the Appendix A.

**Figure 17:**
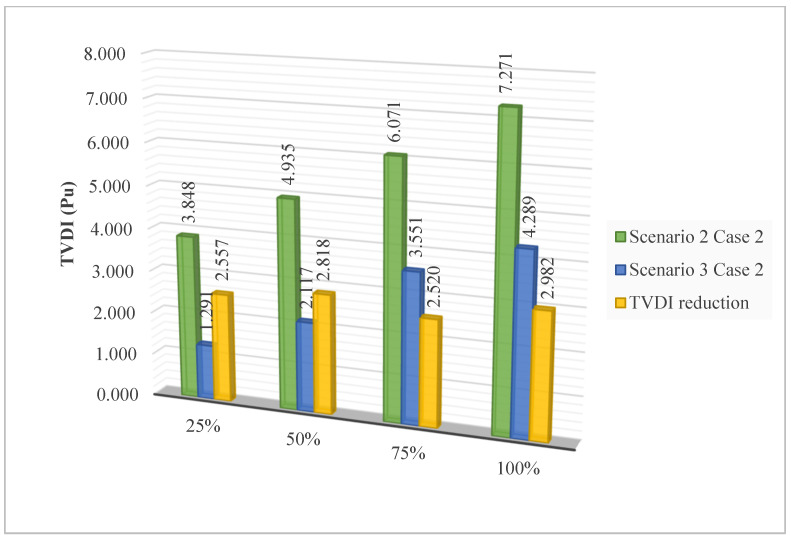
The comparison of TVDI values between Case 2 of Scenario 2 and Case 2 of Scenario 3.

Figure 17 shows the TVDI value achieved in Scenario 2 with the placement of SCs and Scenario 3 with the installation of PVs. Overall, the placement of PVs results in a significant advantage in minimizing the TVDI value compared to SCs in all four levels of EVCS’s penetration. The results in this figure are another proof that demonstrates the effectiveness of placing PVs on the grid compared to SCs. As mentioned previously, the placement of SCs helps increase the voltage value of the grid at the limited area, which sometimes can cause a significant difference from other areas, which suffer a substantial decrease due to the increase in load or particularly the EVCSs in this research. On the other hand, PVs do not affect the voltage profile of the grid as much as SCs do. Therefore, while the penetration of EVCS to the grid becomes more extensive, the voltage value at nodes steadily decreases, with no chaos among different areas within the grid. As a result, the placement of PVs for reaching minimum TVDI is logically better than placing SCs.

**Figure 18:**
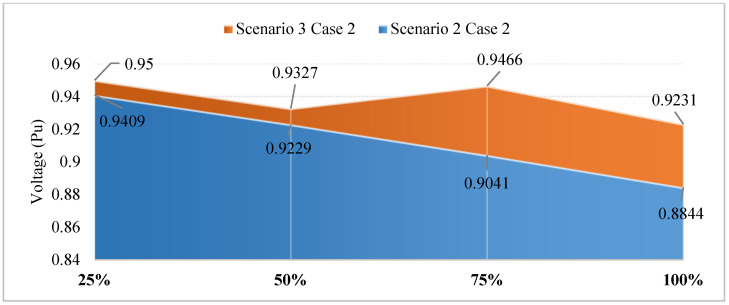
The voltage improvement of Case 2 in Scenario 3 compared to Case 2 in Scenario 2.

Figure 18 presents the voltage values achieved in Scenarios 2 and 3, dealing with the primary objective of minimizing the TVDI value. The observation in this figure indicated that the placement of PVs in this context results in better voltage values than SCs in all EVCS penetration levels. Although PVs do not affect voltage values as much as SCs do, the placement of PVs also leads to an improvement in voltage value, which can be seen at the first penetration of EVCSs. However, by the penetration level of 50% onward, the voltage value is gradually far from the lower limit of 0.95 pu.

#### 4.3.3. The Results Obtained in Case 3

**Figure 19:**
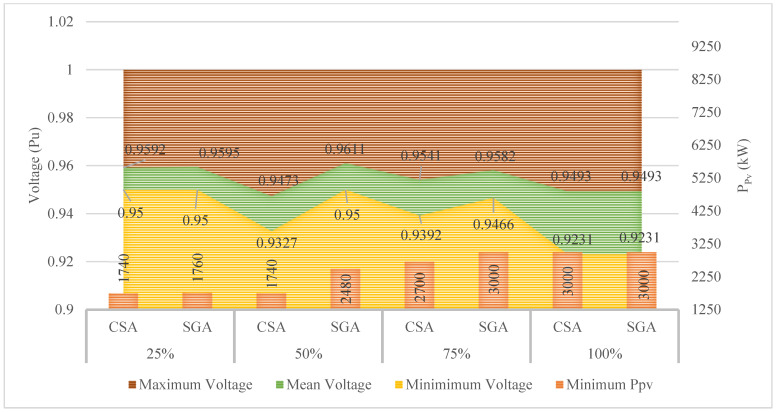
The results obtained by the two algorithms in Case 3 of Scenario 3.

Figure 19 presents the total power output of the three PVs, along with voltage values determined by CSA and SGA in four penetration levels of EVCSs. CSA proves itself to be the better algorithm while resulting in the lower total power output of the three PVs and those found to be SGA in the first three penetration levels. In the last penetration level, both CSA and SGA result in a similar total power output, which is 3000 kW. Regarding the minimum voltage values, the higher power outputs of the three PVs found by SGA lead to better improvement in this regard compared to those of CSA. This phenomenon can be seen in the second and the third penetration levels of EVCs, where SGA determined the minimum voltage values in these levels are 0.95 pu and 0.947 pu, respectively, while those of CSA are only 0.933 pu and 0.939 pu. However, CSA is more effective than SGA in these two penetration levels because the main objective function considered in this case is to minimize the power output of PVs, not maximizing the improvement of voltage. The optimal solution for this case is given in Table A6 of the Appendix A.

### 4.4. The Results Obtained in the Fourth Scenario

#### 4.4.1. The Results Obtained in Case 1

**Figure 20:**
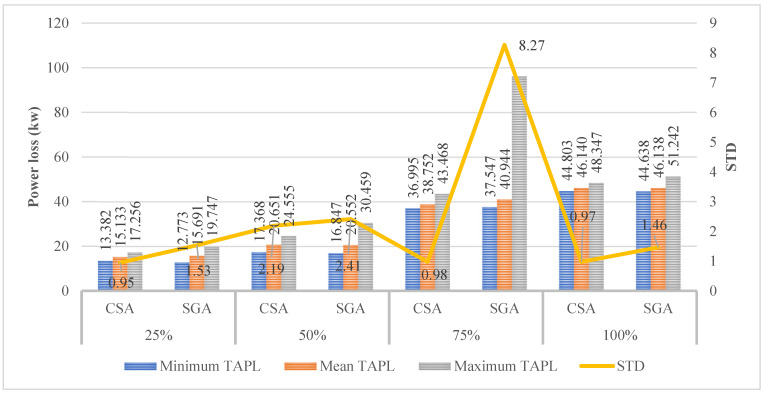
The results obtained by the two algorithms in Case 1 of Scenario 4.

The results shown in Figure 20 indicate that SGA achieves better TPWL values on three penetration levels of EVCSs compared to those of CSA, except for the penetration level of 75%. SGA is particularly better than CSA at 4.55% for the first penetration level, 3% for the second penetration level, and 0.37% for the last. For the third penetration level, CSA reaches a smaller TPWL value, which is 36.995 kW, slightly higher than that of SGA, which is only 37.547 kW and corresponds to 1.47%. Although SGA could surpass CSA in most of the comparison of penetration levels, this method has suffered a higher STD value compared to CSA. This is considered one of the downsides of SGA. The optimal solution for this case is given in Table A7 of the Appendix A.

**Figure 21:**
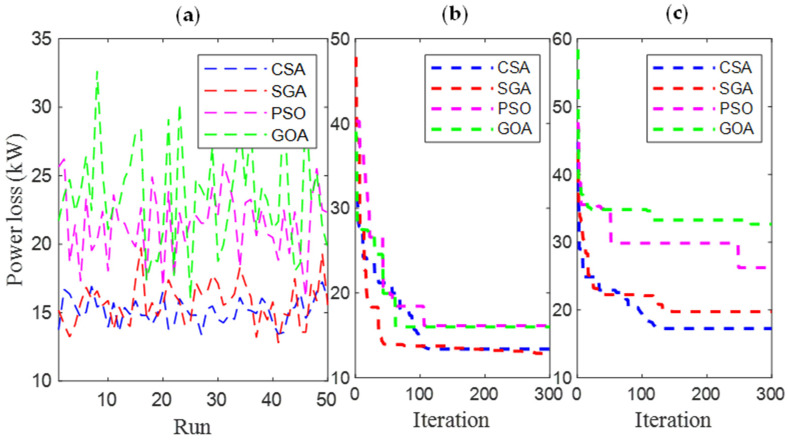
The results of (**a**) 50 test runs, (**b**) the minimum convergences, and (**c**) the maximum convergence obtained by CSA and SGA for 25% penetration of EVCSs in Case 1 of Scenario 4.

**Figure 22:**
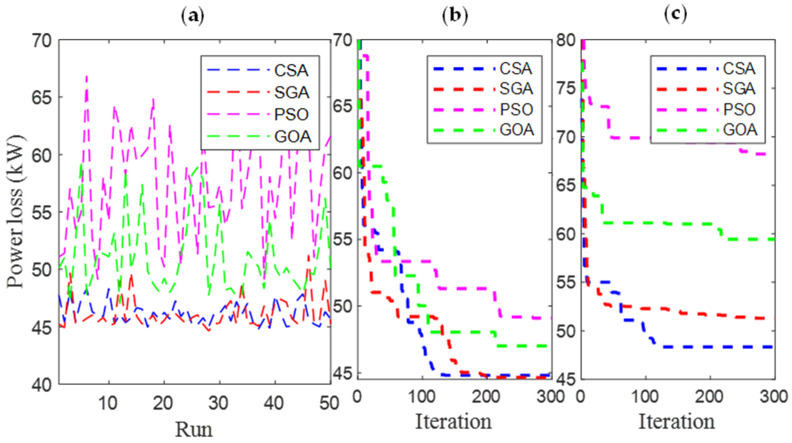
The results of (**a**) 50 test runs, (**b**) the minimum convergences, and (**c**) the maximum convergence obtained by CSA and SGA for 100% penetration of EVCSs in Case 1 of Scenario 4.

Figure 21 and Figure 22 present a detailed comparison of SGA, CSA, PSO [34], and GOA [35] in Cases 1 and 4, with EVCS penetration levels of 25% and 100%, respectively. The performance of all four algorithms is evaluated using various criteria, including the results of 50 test runs (Figure 21a and Figure 22a), minimum convergence (Figure 21b and Figure 22b), and maximum convergence (Figure 21c and Figure 22c). The inclusion of PSO and GOA results provides an intuitive reference, allowing for comparison of the initially selected algorithms with other methods. GOA is a recently published meta-heuristic algorithm, while PSO is widely used and implemented for solving various engineering optimization problems. Observations from Figure 21a and Figure 22a indicate that SGA is the most stable algorithm among the four. Additionally, SGA is the only method to consistently reach the optimal result in its best run, as shown in Figure 21b and Figure 22b, for both EVCS penetration levels (25% and 100%). Clearly, SGA maintains its competitive performance regardless of increasing EVCS penetration. The only observed disadvantage for SGA is its higher maximum power loss values at both EVCS penetration levels, as shown in the maximum convergence plots in Figure 21 and Figure 22c.

**Figure 23:**
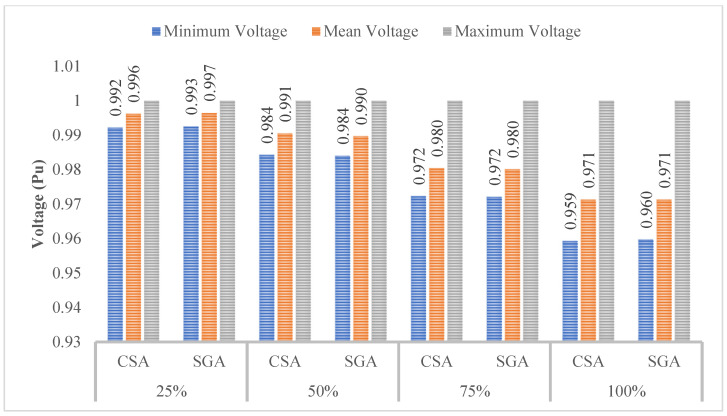
The summary of voltage profile of the grid for each level of EVCS’s penetration in Case 1 of Scenario 4.

The minimum, mean, and maximum voltage values achieved by the two algorithms in the first case of Scenario 4 are presented in Figure 23. The voltage limits are satisfied in all four penetration levels of EVCSs, in which the first penetration level has the highest voltage improvement while the lowest voltage improvement belongs to the last level. In comparison, between the two applied algorithms, SGA offers the highest degree of voltage improvement, which can be viewed in the first and the last penetration levels, while in the remaining levels, SGA and CSA reach a similar minimum voltage.

#### 4.4.2. The Results Obtained in Case 2

**Figure 24:**
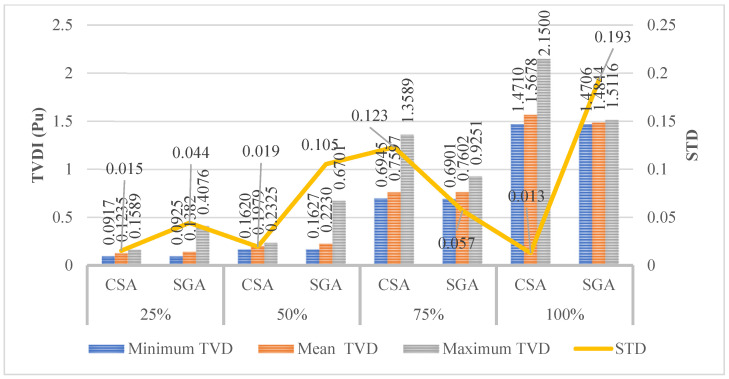
The results obtained by the two algorithms in Case 2 of Scenario 4.

The results shown in Figure 24 clarify that SGA is more effective in finding the best TVDI value in the last two penetration levels of EVCSs, except for the first two. However, the downside of SGA in this case remains the same: the algorithm results in a higher STD value compared to that of CSA in all penetration levels. The optimal solution for this case is given in Table A8 of the Appendix A.

**Figure 25:**
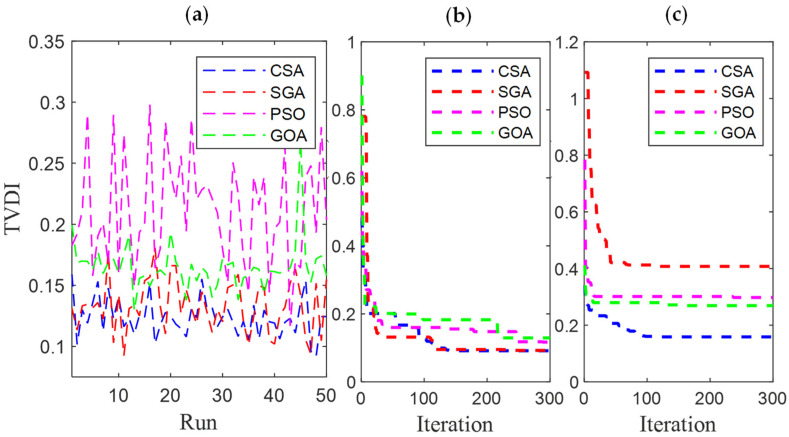
The results of (**a**) 50 test runs, (**b**) the minimum convergences, and (**c**) the maximum convergence obtained by CSA and SGA for 25% penetration of EVCSs in Case 2 of Scenario 4.

Figure 25 presents a detailed comparison of CSA, SGA, GOA, and PSO performance when applied to minimize the total voltage deviation index (TVDI). These figures follow the same layout as Figure 21 and Figure 22. SGA is not the top-performing algorithm at the lowest EVCS penetration of 25%, unlike its performance with the TPWL objective function in the previous subsection. However, as demonstrated in Figure 25a–c, SGA still significantly outperforms GOA and PSO. The best-performing algorithm at 25% penetration is CSA, which exhibits the highest stability across all test runs and the fastest convergence in minimum and maximum convergence, as observed in all three subfigures of Figure 25.

**Figure 26:**
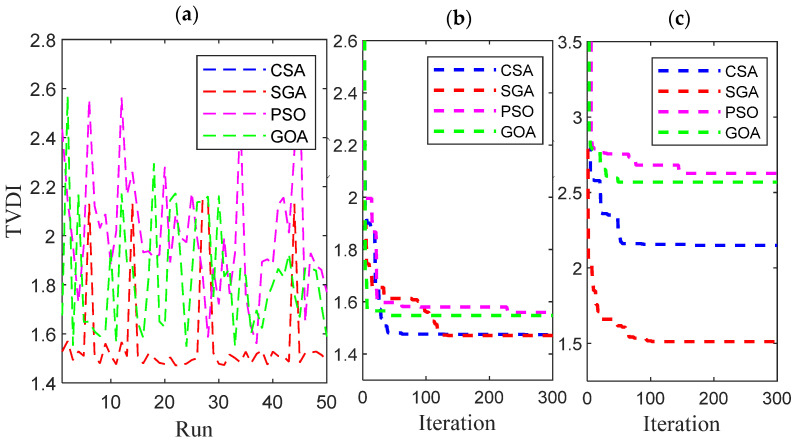
The results of (**a**) 50 test runs, (**b**) the minimum convergences, and (**c**) the maximum convergence obtained by CSA and SGA for 100% penetration of EVCSs in Case 2 of Scenario 4.

Figure 26 displays the results achieved by the four methods using the same criteria as Figure 25 but for the highest EVCS penetration level of 100%. Unlike Figure 25, which indicates CSA as the best-performing algorithm, the results in Figure 26, across all three subfigures, demonstrate that SGA is the most effective algorithm for solving this problem on a larger scale. Specifically, SGA, in this context, exhibits the fastest convergence in both minimum and maximum convergence (presented in Figure 26b,c), along with remarkable stability throughout its 50 test runs (Figure 26a).

**Figure 27:**
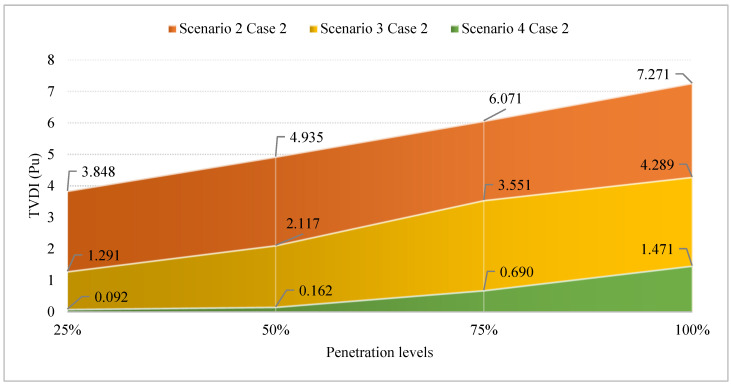
The TVDI values achieved in Case 2 of Scenario 4 compared to the same cases of Scenarios 3 and 2.

**Figure 28:**
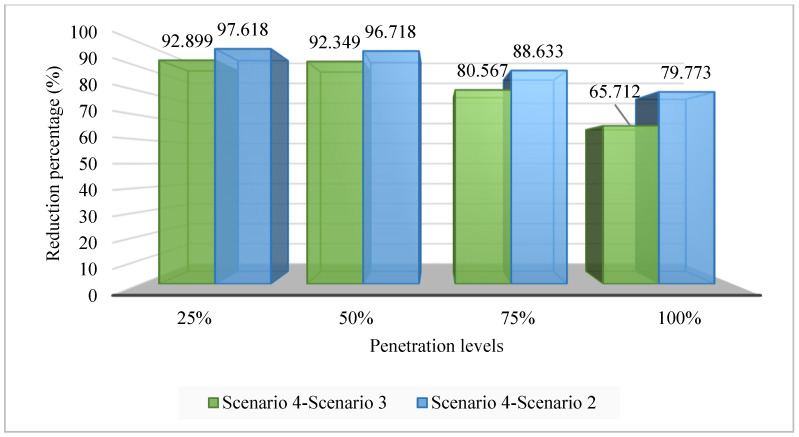
The TVDI reduction of Scenario 4 compared to Scenarios 3 and 2.

Figure 27 presents the TVDI values achieved in Case 2 of Scenario 4 compared to the same case of Scenarios 3 and 2. Clearly, optimizing both SCs and PVs has brought significant efficiency in improving the TVDI value. Clearly, the combination of PVs and SCs mutually mitigates the downsides of each device, providing greater flexibility in reducing the TDVI value across the grid. As mentioned previously, the placement of SCs alone can lead to significant voltage discrepancies between limited grid areas. The addition of PVs alongside SCs enables the regulation and harmonization of active and reactive power flow throughout the grid. This combination is very effective in avoiding the injection of excessive active or reactive power into the grid, which can lead to unstable grid operation and poor efficiency. Note that the data used in the figure to prove the effectiveness of optimizing both SCs and PVs in this case compared to others are based on the best results achieved by the two algorithms at each penetration level. The quantitative reduction of TVDI obtained in Case 2 of Scenario 4 compared to the same cases of both Scenarios 3 and 2 is presented in Figure 28.

**Figure 29:**
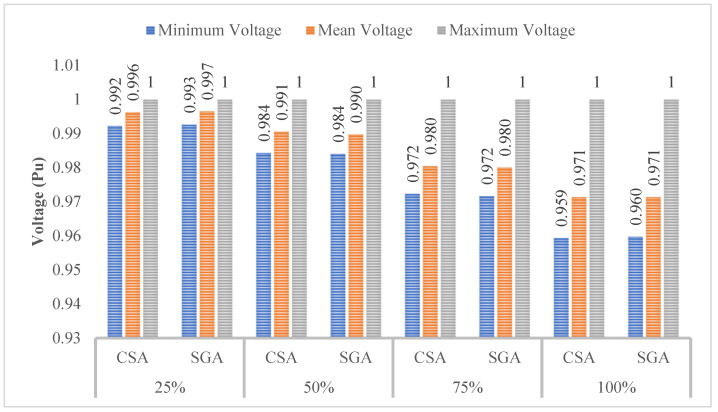
The summary of voltage profile on the grid for each level of penetration in Case 2 of Scenario 4.

The voltage value for each penetration level of EVCSs achieved by CSA and SGA in this case is also given in Figure 29. In the figure, the difference between voltage values achieved by the two algorithms can only be seen in the first and the last penetration levels but not that much, while the other two are the same for both CSA and SGA.

#### 4.4.3. The Results Obtained in Case 3

**Figure 30:**
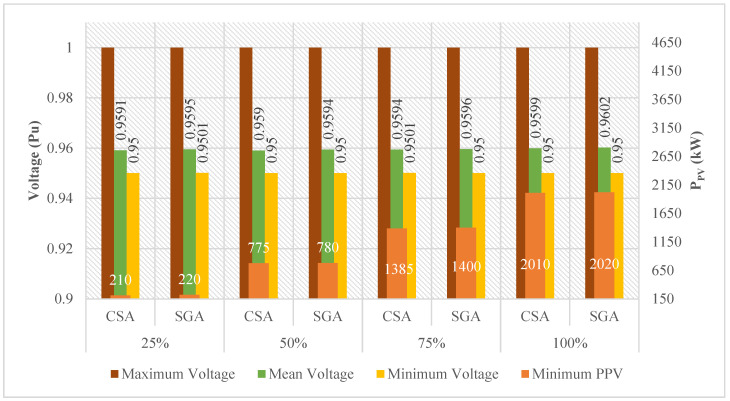
The results obtained by the two algorithms in Case 3 of Scenario 4.

In this case, the CSA and SGA are applied to find the minimum value of the total rated power of all PVs placed on the grid, similar to what has been done in Case 3 of Scenario 4. The results obtained by the two algorithms in this case of Scenario 4 are displayed in Figure 30. Surprisingly, CSA, in this case, can find a better value of the rated power of all PVs than SGA, which is considered the more effective algorithm in previous comparisons. CSA is particularly better than SGA by achieving 775 kW and 1385 kW of the total rated power of all PVs in the second and third penetration levels, while those of SGA are 780 kW and 1400 kW. CSA and SGA perform at the same level in the remaining penetration level. Besides, the minimum voltage values of all penetration levels achieved by the two algorithms are kept in the allowed range, which is 0.95–1.05 pu. The optimal solution for this case is given in Table A9 of the Appendix A.

**Figure 31:**
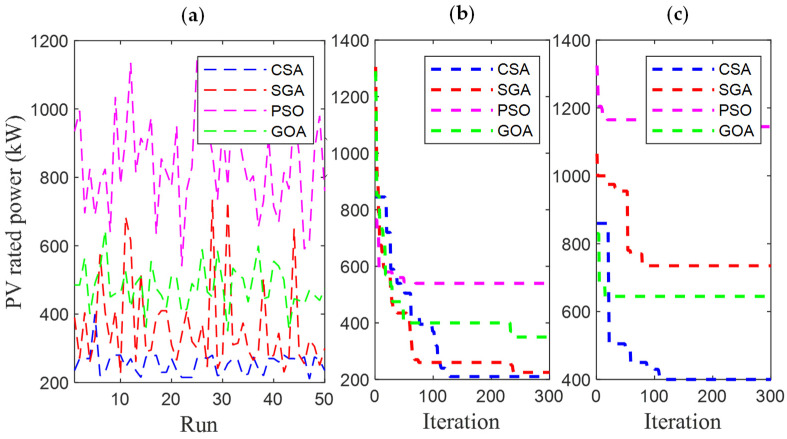
The results of (**a**) 50 test runs, (**b**) the minimum convergences, and (**c**) the maximum convergence obtained by CSA and SGA for 25% penetration of EVCSs in Case 3 of Scenario 4.

**Figure 32:**
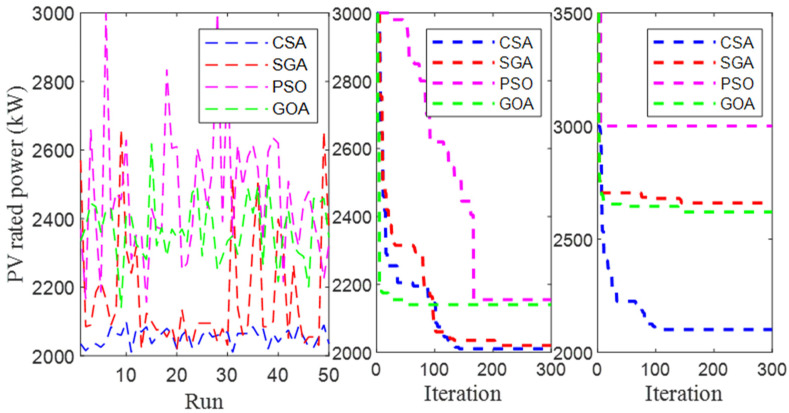
The results of (**a**) 50 test runs; (**b**) the minimum convergences; and (**c**) the maximum convergence obtained by CSA and SGA for 100% penetration of EVCSs in Case 3 of Scenario 4.

Figure 31 and Figure 32 present the results obtained by the four applied algorithms when minimizing the total rated power of all PVs for EVCS penetration levels of 25% and 100%. The results in these figures are presented in the same manner as Figure 25 and Figure 26, using three different criteria: the results of 50 test runs (Figure 31a and Figure 32a), minimum convergence (Figure 31b and Figure 32b), and maximum convergence (Figure 31c and Figure 32c). Observations from all subfigures in Figure 31 and Figure 32 reveal that CSA is the top-performing algorithm among the four. Specifically, CSA achieves the lowest fluctuation among all test runs and the fastest convergence to the optimal value of the objective function in both minimum and maximum convergence. SGA, on the other hand, exhibits high fluctuation and slow convergence, which is particularly evident in Figure 31c and Figure 32c.

**Figure 33:**
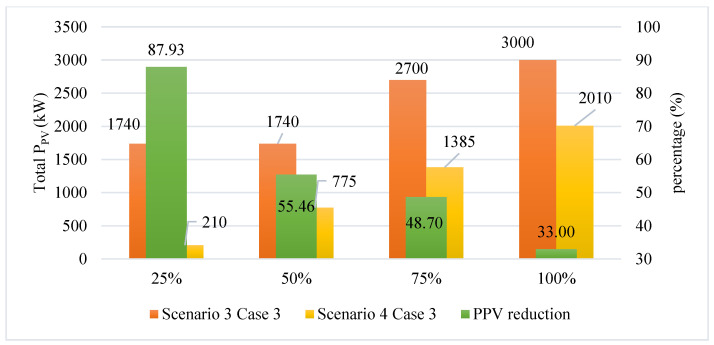
The difference in total PV power output in Case 3 of Scenario 4 compared to a similar case.

Figure 33 compares Case 3 of Scenario 4 and the similar case of Scenario 3 regarding reducing the minimum rated power output of all PVs. Clearly, the combination of SCs and PVs, while being optimized in their allocation, significantly reduced the rated power output of all PVs. In practice, the lower-rated power output of PVs can save more investment costs while implementing the solutions to improve the grid’s operational efficiency.

## 5. Conclusions

This paper successfully applies CSA and SGA to determine the best allocation of EVCSs and other auxiliary devices, such as shunt capacitors and photovoltaic generators, in the IEEE 85-node DEPG. Different scenarios have been implemented while placing EVCSs in the given DEPG to reach the minimum values of three different objective functions, including minimizing the total real power loss (TPWL), minimizing the total voltage deviation index (TVDI), and minimizing the total rated power of photovoltaic generators (PSG). Additionally, the placement of EVCS is limited in particular areas for each charging level. Four penetration levels of EVCSs are evaluated for each objective function, namely 25%, 50%, 75%, and 100%.

The performance of CSA and SGA algorithms varied across objectives and scenarios. For the first objective functions, both CSA and SGA provided similar efficiency while optimally allocating for EVCSs only. In Scenario 2, SGA was better at initial penetration levels, achieving a TPWL value approximately 2kW lower than CSA, but in the last two penetration levels, SGA only performed similarly to CSA. In Scenario 3, SGA outperformed CSA in the last two penetration levels but not the first two. In Scenario 4, except for the third one, SGA was better in three EVCS penetration levels. The second objective was similar to Scenario 2. In Scenario 3, SGA was better at the 40% penetration level but lagged 9% behind CSA at the highest level. In Scenario 4, SGA was superior in the last two penetration levels. For the third objective, SGA consistently underperformed CSA in Scenarios 3 and 4, with SGA’s total rated power output consistently higher than CSA’s. Regarding the voltage profile, both algorithms showed similar trends. The case with EVCSs only resulted in voltage far below the limit, improved by adding auxiliary devices in Scenarios 2 and 3. Scenario 4, combining SCs and PVs, provided the best voltage improvement, meeting all constraints.

This study significantly contributes to engineering and academic fields by addressing DEPG planning and expansion. From an engineering perspective, the research analyzes the impact of EVCS integration and proposes solutions to mitigate adverse effects by optimizing the placement of SCs and PVs separately and combined. In terms of the computing method, the study provides a detailed performance evaluation of CSA and SGA algorithms, using charts and figures to demonstrate that SGA excels in solving the first two objective functions. In contrast, CSA demonstrates superior performance in addressing the third objective function.

Besides the results and achievements as stated above, the study also has several limitations that must be improved for better comprehensiveness, as follows:

- The study only focuses on solving the considered problem in the aspect of planning and expanding the current DEPG, while the operational process is not considered.
- The study only applies the geographical constraints when placing EVCSs, and these constraints are not applied when placing other auxiliary devices.
- The penetration of renewable energy sources such as PVs is not thoroughly evaluated and assessed by using real data.
- SGA is proven to be the better algorithm in two out of three objective functions; however, the method is unstable due to high STD value, especially while dealing with the larger scale of the considered problem with a high penetration level of EVCSs and a large number of desired control variables.

## Figures and Tables

**Table 1:** The description of all scenarios conducted in the research.

Scenario	Auxiliary Devices	Case Study	Main Objective Function	Number of Control Variables	Presets (*N_Ps_*–*HI*)	
					CSA	SGA
1	None	1	Minimizing the total active power loss	3	20–50	40–50
2	SCs	1	Minimizing the total active power loss	6	30–200	60–200
		2	Minimizing total voltage deviation	6	30–200	60–200
3	03 PVs	1	Minimizing the total active power loss	6	30–200	60–200
		2	Minimizing total voltage deviation	6	30–200	60–200
		3	Minimizing total PV’s rated power	6	30–200	60–200
4	3PVs + 3SCs	1	Minimizing the total active power loss	12	50–300	100–300
		2	Minimizing total voltage deviation	12	50–300	100–300
		3	Minimizing total PV’s rated power	12	50–300	100–300

**Table 2:** The EVCSs’ penetration for each scenario.

Scenario	The Penetration of EVCSs (%)	Number of EVCSs for Each Level
1, 2, 3, 4	25	4LV1–4LV2–2LV3
	50	8LV1–8LV2–4LV3
	75	12LV1–12LV2–6LV3
	100	16LV1–16LV2–8LV3

**Table 3:** The specifications of EVCS and other auxiliary devices.

Devices	Nominated Active Power (kW)	Nominated Reactive Power (kVAr)	Step Size
EVCS–LV1 [32]	30	0	0
EVCS–LV2 [32]	60	0	0
EVCS–LV3 [32]	150	0	0
PVs	1000	0	5 kW
SCs	0	900	30 kVAr

## Data Availability

Data of the IEEE 85-node system are available in [33].

## Nomenclature

The following abbreviations are used in this manuscript:

$P_{Load,n}$	The active power of load at node *n*
${No}_{LV1},\;{No}_{LV2}$, ${No}_{LV3}$	Number of installed level 1, level 2 and level 3 EVCSs
$P_{LV1}$, $P_{LV2}$ and $P_{LV3}$	Active power demand of each Level-1, Level-2 and Level-3 EVCS
$Q_{grid}$	Reactive power supplied by the grid
${No}_{cb}$	Number of all installed capacitor banks
$Q_{cb,c}$	Capacity of the *cth* installed capacitor bank
$Q_{Load,n}$	Reactive power deamand of the load at node *n*
${Reac}_{dl}$	Reactance of the *dlth* distribution line
$Q_{CB}^{min}$, $Q_{CB}^{max}$	The minimum and maximum reactive power supplied by the SC *c*
${Lo}_{pv,y}$	Location of the *yth* installed PV system
${Lo}_{LV1},\;{Lo}_{LV2},\;{Lo}_{LV3}$	Location of Type-1, Type-2 and Type-3 EVCSs.
${SLo}_{LV1},\;{SLo}_{LV2},\;{SLo}_{LV3}$	Location set of Type-1, Type-2 and Type-3 EVCSs
$S_{n}^{new\_s1}$	The newly updated individual *n* in Stage 1
*N_Ps_*	The initial population size
$S_{n}$	The *nth* current solution
${ef}_{1},$${ef}_{2}$	Exploration factors manipulating the exploration capabilities
$S_{PB,n}$	The so-far best location of the individual *n*
$S_{GB}$	The best location of the whole population
$\omega_{1}$, $\omega_{2}$, $\omega_{3}$	Random factors between zero and one
rf	Reference coefficient determining the method of updating a particular individual
δ, α, β	The parameters that used to manipulate the exploitation capabilities
${Ir}_{h},\;{Ir}_{H}$	The current and the highest indexes of the iteration
${ub}_{n},\;{lb}_{n}$	Upper and lower location bounds for all individuals
rand	Random values between zero and one
$V_{n}$, $V_{n}^{new}$	The current and new velocities of the *nth* individual
$S_{n}^{new\_s2}$	The new location updated in Stage 2 for the individual *n*
${IF}_{1}$, ${IF}_{2}$	The influence factors
$\omega_{4}$, $\omega_{5}$	Random factor between zero and one
${AF}_{1}$, ${AF}_{2}$, ${AF}_{3}$	Adaptive factors
Rf	Regulation term
${Ft}_{n}$	Fitness value of the individual *n*
$S_{WS}$	The worst individual evaluated by its fitness function
$\omega_{6}$	The random factor and its value is between zero and one
BR	The value determined by the Brownian distribution

## Appendix A

**Table A1:** The optimal solution achieved by CSA and SGA in the first scenario.

Penetration Level	25%		50%		75%		100%	
Method	CSA	SGA	CSA	SGA	CSA	SGA	CSA	SGA
Minimum Power loss (kW)	307.3848461	307.3848461	419.0020249	419.0020249	562.9795245	562.9795245	744.053316	744.053316
${SLo}_{LV1}$ (node)	33	33	33	33	33	33	33	33
${SLo}_{LV2}$ (node)	9	9	9	9	9	9	9	9
${SLo}_{LV3}$ (node)	65	65	65	65	65	65	65	65

**Table A2:** The optimal solution achieved by CSA and SGA in Case 1 of Scenario 2.

Penetration Level	25%		50%		75%		100%	
Method	CSA	SGA	CSA	SGA	CSA	SGA	CSA	SGA
Minimum Power loss (kW)	251.0882	249.0995	347.2369	347.2369	472.3812	472.3812	648.4134	648.4134
${SLo}_{LV1}$ (node)	34	34	34	34	34	34	34	34
${SLo}_{LV2}$ (node)	10	10	10	10	10	10	10	10
${SLo}_{LV3}$ (node)	66	66	66	66	66	66	66	66
${No}_{cb1}$ (node)	70	44	69	34	48	34	52	52
${No}_{cb2}$ (node)	44	52	52	52	69	69	70	44
${No}_{cb3}$ (node)	52	69	34	69	34	48	44	70
$Q_{cb,1}$ (kVar)	840	900	840	900	900	900	870	870
$Q_{cb,1}$ (kVar)	900	900	900	900	840	840	900	870
$Q_{cb,3}$ (kVar)	900	840	900	840	900	900	870	900

**Table A3:** The optimal solution achieved by CSA and SGA in Case 2 of Scenario 2.

Penetration Level	25%		50%		75%		100%	
Method	CSA	SGA	CSA	SGA	CSA	SGA	CSA	SGA
Minimum TVDI (Pu)	3.8481	3.8481	4.9349	4.9349	6.0713	6.0713	7.2707	7.2707
${SLo}_{LV1}$ (node)	33	33	33	33	33	33	33	33
${SLo}_{LV2}$ (node)	9	9	9	9	9	9	9	9
${SLo}_{LV3}$ (node)	65	65	65	65	65	65	65	65
${No}_{cb1}$ (node)	68	68	51	33	47	68	69	43
${No}_{cb2}$ (node)	51	51	33	51	33	47	51	51
${No}_{cb3}$ (node)	43	43	68	68	68	33	43	69
$Q_{cb,1}$ (kVar)	840	840	900	900	900	840	900	900
$Q_{cb,1}$ (kVar)	900	900	900	900	900	900	870	840
$Q_{cb,3}$ (kVar)	900	900	840	840	840	900	870	900

**Table A4:** The optimal solution achieved by CSA and SGA in Case 1 of Scenario 3.

Penetration Level	25%		50%		75%		100%	
Method	CSA	SGA	CSA	SGA	CSA	SGA	CSA	SGA
Minimum Power loss (kW)	112.2373	112.2373	128.9000	128.9948	168.3862	146.6976	264.5539	332.2845
${SLo}_{LV1}$ (node)	33	33	33	33	33	33	33	33
${SLo}_{LV2}$ (node)	9	9	9	9	9	9	9	9
${SLo}_{LV3}$ (node)	65	65	65	65	65	65	65	65
${Lo}_{pv,1}$ (node)	33	33	33	64	51	64	44	43
${Lo}_{pv,2}$ (node)	8	63	57	33	67	47	69	48
${Lo}_{pv,3}$ (node)	63	8	64	57	64	62	48	70
$P_{pv,1}$ (kW)	750	750	930	920	900	1000	620	895
$P_{pv,2}$ (kW)	990	870	775	1000	900	1000	950	810
$P_{pv,3}$ (kW)	870	990	915	700	900	1000	1000	945

**Table A5:** The optimal solution achieved by CSA and SGA in Case 2 of Scenario 3.

Penetration Level	25%		50%		75%		100%	
Method	CSA	SGA	CSA	SGA	CSA	SGA	CSA	SGA
Minimum TVDI (Pu)	1.2908	1.2908	2.1171	2.1171	3.9041	3.5515	4.2890	4.3085
${SLo}_{LV1}$ (node)	33	33	33	33	33	33	33	33
${SLo}_{LV2}$ (node)	9	9	9	9	9	9	9	9
${SLo}_{LV3}$ (node)	65	65	65	65	65	65	65	65
${Lo}_{pv,1}$ (node)	47	40	44	51	51	64	33	69
${Lo}_{pv,2}$ (node)	40	47	51	44	67	62	31	34
${Lo}_{pv,3}$ (node)	69	69	69	69	64	47	69	29
$P_{pv,1}$ (kW)	1000	1000	1000	1000	900	1000	1000	1000
$P_{pv,2}$ (kW)	1000	1000	1000	1000	900	1000	1000	1000
$P_{pv,3}$ (kW)	1000	1000	1000	1000	900	1000	1000	1000

**Table A6:** The optimal solution achieved by CSA and SGA in Case 3 of Scenario 3.

Method	CSA	SGA	CSA	SGA	CSA	SGA	CSA	SGA
Minimum P_PV_ (kW)	1740	1760	1740	2480	2700	3000	3000	3000
${SLo}_{LV1}$ (node)	33	33	33	33	33	33	33	33
${SLo}_{LV2}$ (node)	9	9	9	9	9	9	9	9
${SLo}_{LV3}$ (node)	65	65	65	65	65	65	65	65
${Lo}_{pv,1}$ (node)	50	47	47	67	51	47	29	34
${Lo}_{pv,2}$ (node)	67	84	67	84	64	62	69	29
${Lo}_{pv,3}$ (node)	83	69	77	48	67	64	34	69
$P_{pv,1}$ (kW)	885	855	940	1000	900	1000	1000	1000
$P_{pv,2}$ (kW)	755	170	995	480	900	1000	1000	1000
$P_{pv,3}$ (kW)	100	735	545	1000	900	1000	1000	1000

**Table A7:** The optimal solution achieved by CSA and SGA in Case 1 of Scenario 4.

Penetration	25%		50%		75%		100%	
Method	CSA	SGA	CSA	SGA	CSA	SGA	CSA	SGA
Minimum TAPL	13.3818	12.7733	17.3678	16.8469	36.9955	37.0223	44.8032	44.6377
${SLo}_{LV1}$ (node)	33	33	33	33	33	33	33	33
${SLo}_{LV2}$ (node)	9	9	9	9	9	9	9	9
${SLo}_{LV3}$ (node)	65	65	65	65	65	65	65	65
${No}_{cb1}$ (node)	33	33	8	8	33	24	63	33
${No}_{cb2}$ (node)	10	66	33	66	10	33	11	66
${No}_{cb3}$ (node)	63	9	66	34	63	66	32	8
$Q_{cb,1}$ (kVar)	750	750	840	900	750	840	750	720
$Q_{cb,1}$ (kVar)	630	630	690	630	630	630	480	570
$Q_{cb,3}$ (kVar)	690	720	630	600	690	630	870	900
${Lo}_{pv,1}$ (node)	32	63	32	64	57	64	32	32
${Lo}_{pv,2}$ (node)	8	32	56	32	32	57	64	63
${Lo}_{pv,3}$ (node)	66	8	64	8	64	32	63	64
$P_{pv,1}$ (kW)	895	900	1000	1000	1000	1000	1000	1000
$P_{pv,2}$ (kW)	1000	870	1000	1000	1000	1000	1000	1000
$P_{pv,3}$ (kW)	840	1000	1000	1000	1000	1000	1000	1000

**Table A8:** The optimal solution achieved by CSA and SGA in Case 2 of Scenario 4.

Penetration	25%		50%		75%		100%	
Method	CSA	SGA	CSA	SGA	CSA	SGA	CSA	SGA
Minimum TVDII (Pu)	0.0917	0.0925	0.1620	0.1627	0.6945	0.6901	1.4710	1.4706
${SLo}_{LV1}$ (node)	33	33	33	33	33	33	33	33
${SLo}_{LV2}$ (node)	9	9	9	9	9	9	9	9
${SLo}_{LV3}$ (node)	65	65	65	65	65	65	65	65
${No}_{cb1}$ (node)	51	4	26	30	47	66	68	43
${No}_{cb2}$ (node)	24	38	47	60	40	40	43	80
${No}_{cb3}$ (node)	79	57	82	68	71	44	81	68
$Q_{cb,1}$ (kVar)	690	900	900	900	900	840	900	900
$Q_{cb,1}$ (kVar)	720	810	840	840	900	900	900	840
$Q_{cb,3}$ (kVar)	810	900	900	900	840	900	840	900
${Lo}_{pv,1}$ (node)	56	71	62	64	67	67	47	40
${Lo}_{pv,2}$ (node)	32	33	32	11	34	47	40	47
${Lo}_{pv,3}$ (node)	66	10	66	34	65	76	69	69
$P_{pv,1}$ (kW)	955	990	1000	1000	1000	1000	1000	1000
$P_{pv,2}$ (kW)	885	995	1000	1000	1000	1000	1000	1000
$P_{pv,3}$ (kW)	1000	1000	1000	1000	1000	1000	1000	1000

**Table A9:** The optimal solution achieved by CSA and SGA in Case 3 of Scenario 4.

Penetration	25%		50%		75%		100%	
Method	CSA	SGA	CSA	SGA	CSA	SGA	CSA	SGA
Minimum P_PV_ (kW)	210	220	775	780	1385	1400	2010	2020
${SLo}_{LV1}$ (node)	33	33	33	33	33	33	33	33
${SLo}_{LV2}$ (node)	9	9	9	9	9	9	9	9
${SLo}_{LV3}$ (node)	65	65	65	65	65	65	65	65
${No}_{cb1}$ (node)	43	67	33	33	67	67	65	64
${No}_{cb2}$ (node)	67	43	65	66	65	76	43	32
${No}_{cb3}$ (node)	51	47	67	63	51	53	73	84
$Q_{cb,1}$ (kVar)	870	900	900	840	900	900	900	900
$Q_{cb,1}$ (kVar)	900	840	870	900	870	840	870	900
$Q_{cb,3}$ (kVar)	870	900	870	900	870	900	870	840
${Lo}_{pv,1}$ (node)	41	70	35	75	79	84	13	73
${Lo}_{pv,2}$ (node)	64	32	48	52	34	78	49	48
${Lo}_{pv,3}$ (node)	68	33	67	84	71	34	66	76
$P_{pv,1}$ (kW)	20	175	545	250	85	105	180	670
$P_{pv,2}$ (kW)	35	35	5	530	700	605	855	805
$P_{pv,3}$ (kW)	155	10	225	0	600	690	975	545
